# Dexmedetomidine vs. Propofol-Fentanyl for Bispectral Index (BIS)-Guided Sedation During Awake Fiberoptic Intubation in Neurosurgical Patients With Anticipated Difficult Airways: An Observational Study

**DOI:** 10.7759/cureus.105150

**Published:** 2026-03-13

**Authors:** Madhusudhana Rao Bathala, Manikandan Sethuraman, Soumya Madhusudan, Archana Turimella, Seham Syeda, Geetha Lakshminarasimhaiah, Smita Musti, Saurabh Bhandge

**Affiliations:** 1 Anesthesiology/Neuroanesthesia and Neurocritical Care, Ramaiah Medical College, Ramaiah University of Applied Sciences, Bengaluru, IND; 2 Neuroanesthesia and Neurocritical Care, Sree Chitra Tirunal Institute for Medical Sciences and Technology, Thiruvananthapuram, IND; 3 Anesthesiology, St. John’s Medical College, Bengaluru, IND; 4 Emergency Medicine, Savithramma Clinic, Bengaluru, IND; 5 Anesthesiology, Yenepoya Medical College, Mangalore, IND; 6 Anesthesiology, Shri Rawatpura Sarkar Institute of Medical Sciences and Research, Raipur, IND

**Keywords:** anticipated difficult airways, awake fiberoptic intubation, bispectral index, conscious sedation, dexmedetomidine, propofol and fentanyl, spray-as-you-go (saygo) technique

## Abstract

Introduction

Awake fiberoptic intubation (AFOI) is commonly employed in neurosurgical patients with anticipated difficult airways to minimize spinal cord compression and intracranial pressure fluctuations, although procedural discomfort remains a major limitation. This prospective observational study evaluated the AFOI with two commonly used conscious sedation regimens-dexmedetomidine and a propofol-fentanyl combination-guided by Bispectral Index (BIS) and the Observer’s Assessment of Alertness/Sedation (OAA/S) scale, along with spray-as-you-go (SAYGO) topical anesthesia. Outcomes assessed included AFOI conditions - cough severity and intubation conditions; time to achieve target sedation (BIS value of 70); hemodynamic changes; total intubating attempts; intubation time; correlation between BIS and OAA/S; perioperative complications; and postoperative recall.

Methods

Forty consecutive adult patients undergoing AFOI for elective neurosurgical procedures received conscious sedation with either dexmedetomidine (Group D) or propofol-fentanyl (Group PF) as part of routine clinical practice. Conscious sedation was titrated using BIS, and the corresponding OAA/S scores were assessed. Airway topicalization was achieved using the SAYGO technique.

Results

Patients receiving propofol-fentanyl demonstrated superior intubation conditions compared with dexmedetomidine, as evidenced by higher total intubation scores (7.80 ± 0.62 vs. 7.11 ± 1.24; p = 0.039) and shorter intubation times (16.10 ± 10.95 vs. 27.79 ± 21.18 seconds; p = 0.036). Additionally, the propofol-fentanyl group also exhibited less coughing, better endotracheal tube tolerance, lower lignocaine requirements, and improved postoperative recovery profile with fewer complaints of sore throat during the first 48 hours after surgery. Procedure recall was absent in both groups.

Conclusion

In routine clinical practice, BIS-guided conscious sedation with a propofol-fentanyl combination was associated with better intubating conditions and procedural tolerance than dexmedetomidine during AFOI in high-risk neurosurgical patients. The SAYGO technique further enhanced patient comfort and safety, providing a practical alternative to conventional airway nerve blocks.

## Introduction

Awake fiberoptic intubation (AFOI) is widely used for the management of anticipated difficult airways, particularly in neurosurgical patients, as it helps preserve cervical spine stability and minimizes fluctuations in intracranial pressure [1]. Despite adequate topical airway anesthesia, AFOI is frequently associated with significant patient discomfort, including gagging, coughing, and pain, which may result in postoperative sore throat, exacerbate cervical cord compression [2], or increase intracranial pressure [3]. Although newer terminology such as “awake flexible scope intubation” (Awake FSI) [4] is increasingly used, the term “awake fiberoptic bronchoscopy intubation (AFOB)” [5,6] is retained in this manuscript for consistency with established guidelines and prevailing indexed terminology.

Conscious sedation is a controlled drug-induced state in which patients remain responsive to verbal commands or light tactile stimulation, improving tolerance while preserving spontaneous ventilation and airway patency [7,8]. An ideal sedation regimen for AFOI should provide anxiolysis, analgesia, and amnesia, allow titration, and minimize respiratory and cardiovascular adverse effects [2]. Dexmedetomidine provides sedation and analgesia with minimal respiratory depression, whereas propofol-fentanyl sedation is widely used and may offer favorable intubating conditions but carries a risk of oversedation and respiratory compromise [9,10]. Airway topicalization can be achieved using regional nerve blocks or the less invasive spray-as-you-go (SAYGO) technique [11].

While several studies have evaluated sedative agents and intubation conditions during AFOI in patients with difficult airways, limited data are available on the comparative performance of commonly used sedation regimens when guided by objective depth-of-sedation monitoring. In routine clinical practice, monitoring with the Bispectral Index (BIS) [12], in conjunction with validated subjective clinical sedation scales such as the OAA/S scale [12], may enhance procedural safety and patient comfort.

This study was conducted with the primary objective to assess AFOI conditions - cough severity and intubation conditions; time to achieve target sedation (BIS-70). The secondary outcomes were to evaluate the hemodynamic changes, total intubation attempts, intubation time, BIS values, and corresponding changes in OAA/S, comparison between the difficult airway and the intubation conditions, and the need for additional airway maneuvers, postoperative airway complications like sore throat, and postoperative recall during the 48 hours postoperatively.

## Materials and methods

Study design

This study was conducted as a prospective, open-label, observational comparative study. Ethical approval was obtained from the Technical Advisory Committee and the Institutional Ethics Committee of Sree Chitra Tirunal Institute for Medical Sciences and Technology, Thiruvananthapuram (SCT/IEC/626/June-2014), a tertiary-care, university-level referral center. Written informed consent was obtained from all participants in English or Malayalam (regional language), including consent for publication. Data were collected prospectively over one year from June 2014 to June 2015. All patient data were anonymized and de-identified before the analysis.

A total of 40 consecutive adult patients aged 19-60 years belonging to the American Society of Anesthesiologists (ASA) physical status I-II with anticipated difficult airways were included. Exclusion criteria were patient’s denial, ASA status ≥III, GCS score <15, history of stroke or cerebral aneurysm, emergency or redo surgery, hypersensitivity to the study drugs, preoperative bradycardia (heart rate <50 beats/minute), heart block, uncontrolled hypertension, coronary artery disease, hepatic or renal dysfunction, pregnancy or lactation, infection or sepsis, hemodynamic instability, or on inotropic support.

An anticipated difficult airway was defined in accordance with the ASA and Difficult Airway Society (DAS) guidelines as the presence of predictors of difficult facemask ventilation and/or difficult tracheal intubation. Predictors of difficult tracheal intubation included a history of previous difficult intubation, reduced thyromental distance (TMD) (<6 cm), limited mouth opening (MO) (inter-incisor distance <3 cm), and restricted head and neck movement (<80°) using a goniometer. Predictors of difficult facemask ventilation were modified Mallampati class (MMP) (≥II) and the presence of obstructive sleep apnea [5,6,13-16].

All patients received intramuscular glycopyrrolate 0.2 mg 30 minutes before transfer to the operating room (OR) [6]. In the OR, only essential personnel were present to minimize patient discomfort. Standard ASA monitoring, including five-lead electrocardiography, non-invasive blood pressure, pulse oximetry (Philips Intellivue MX700, Philips Healthcare, Andover, MA), and bispectral index monitoring (Aspect A-2000, Soma Tech Intl, Bloomfield, CT), was instituted, and baseline values were recorded. After securing intravenous access and initiating isotonic saline at 5-10 mL/kg, supplemental oxygen at 6 L/minute was administered via face mask until completion or abandonment of intubation, with the head maintained in a neutral position. Topical airway anesthesia was achieved using five intraoral sprays of 10% lidocaine followed by the SAGO technique with intermittent 1-2 mL boluses of 2% lignocaine via the suction port or working channel of a FOB as it is advanced toward the trachea [11]. The total lignocaine dose was limited to 9 mg/kg [6].

Patients received conscious sedation using one of the two regimens: dexmedetomidine (Group D) or a propofol-fentanyl combination (Group PF). The choice of sedative regimen was made by the attending neuroanesthesiologist, not involved in performing AFOI, based on institutional practice and patient-specific factors, such as age, comorbidities, airway assessment, while ensuring comfort, spontaneous ventilation, and neurological responsiveness. Blinding was not feasible due to the nature of the interventions and subjective selection of sedation regimens, representing a potential source of selection bias.

In Group D, dexmedetomidine (DEXEM®, Themis Chemicals, Haridwar, India) was administered as a loading dose of 1 µg/kg over 10 minutes [16] followed by a maintenance infusion of 0.5 µg/kg/hour [16]. While in Group PF, propofol (NEOROF®, Neon Laboratories, Mumbai, India) was infused at 1 mg/kg/hour in combination with fentanyl (TROFENTYL®, Troikaa Pharmaceuticals Ltd., Gujarat, India), administered as a 1 µg/kg bolus over 10 minutes followed by a maintenance infusion of 1 µg/kg/hour [16]. In both groups, sedation was titrated to achieve a BIS value of approximately 70 before intubation, with the OAA/S score recorded concurrently to provide a clinical assessment of sedation depth.

Once the target sedation depth of BIS 70 was attained, a bite block was inserted, orotracheal flexible FOB (Karl Storz FIVE 6.5 Flexible Intubation Video Endoscope, KARL STORZ Endoscopy, Tuttlingen, Germany) connected to the C-MAC® monitor for real-time visualization and guidance was then performed. To minimize inter-operator variability, all AFOIs were performed by a single neuroanesthesiologist with formal training and experience in difficult airway management, ensuring uniform application of the intubation technique across the study population.

An appropriately sized endotracheal tube (ETT) was railroaded over the flexible intubation videoendoscope and advanced into the trachea and positioned approximately 3-4 cm above the carina. Placement of the ETT was also confirmed by capnography and bilateral chest auscultation.

Baseline heart rate, blood pressure, oxygen saturation (SpO2), BIS, and OAA/S scores were recorded before sedation, at one minute, every three minutes until completion of intubation, and every five minutes thereafter for 30 minutes. Critical events like hemodynamic fluctuation ± 20% from baseline and apneic episodes were documented and managed according to standard clinical practice. Apnea lasting more than 60 seconds or SpO2 below 95% was managed by reducing the infusion rate to half and providing bag-mask ventilation with 100% oxygen. In the event of persistent apnea lasting more than two minutes, the sedative infusion was temporarily withheld until spontaneous respiration resumed.

Cough severity was graded using a standardized scoring system (1 = no cough; 2 = mild cough (<3 episodes); 3 = moderate cough (three to five episodes); 4 = severe cough (>5 episodes)) (presented in a figure under Results) [17-19]. Intubation conditions [20] were assessed using an established intubation scoring system, which is based on three criteria consisting of anesthesia quality, vocal cords (VC) relaxation, and immediate tracheal tube tolerance. Anesthesia quality comprised (asleep, deep sedation: score 2; if slight, resistance: score 0), VC relaxation (if no relaxation of VC: score 0; if VC are in part, middle: score 1; if VC are relaxed, open: score 2) and immediate tracheal tube tolerance (bad, disturbing - cough, swallowing: score 0; middle; coughing or swallowing not disturbing the procedure: score 2; good, no coughing or swallowing: score 4). If the total Intubation score < 3: difficult or impossible fiberoptic intubation; between 3 and 7: disturbed procedure for fiberoptic intubation; successful tracheal intubation, and if the total score was more than 7: easy fiberoptic intubation; successful tracheal intubation (presented in the following tables under Results).

Additional parameters recorded included time to achieve target BIS and OAA/S scores, intubation duration, number of attempts, and the need for additional airway maneuvers, infusion duration, total doses of sedative agents, and lignocaine.

Intubation time was defined as the interval from insertion of the FOB into the oral cavity (after initial airway topicalization) to confirmation of correct ETT placement. Criteria for abandoning AFOI included patient refusal, severe coughing (score 4), significant resistance, hemodynamic instability (symptomatic bradycardia unresponsive to treatment or blood pressure variation ± 20%), SpO2 below 90%, inability to achieve adequate sedation (BIS ≈70), or more than three intubation attempts. In these situations, AFOI was discontinued, and tracheal intubation was completed under general anesthesia.

Following intubation, patients underwent a brief neurological assessment, including response to simple verbal commands. Subsequent anesthesia management proceeded according to the discretion of the attending neuroanesthesiologist. Postoperatively, patients were evaluated for 48 hours for any throat discomfort related to intubation and recall of the procedure.

Statistical analysis

Data were compiled using Microsoft Excel 2019 (Microsoft Corporation, Redmond, WA) and analyzed using SPSS Statistics for Windows, version 17.0 (SPSS Inc., Chicago, IL). Based on previous studies [4,21,22], the minimum required sample size was 17 patients per group; allowing for a 10% attrition rate, 40 patients (20 per group) were included, providing 80% power at a two-sided alpha level of 0.05. Statistical analysis was performed using appropriate parametric and non-parametric tests.

Continuous variables were tested for normality using the Shapiro-Wilk test. Normally distributed data are presented as mean ± standard deviation (SD), whereas skewed or ordinal data are expressed as median with interquartile range (IQR). Categorical variables are summarized as frequencies and percentages.

Between-group comparisons were performed as follows:

Normally distributed continuous variables (e.g., age, weight, height, BMI, baseline hemodynamic parameters, and baseline BIS) were analyzed using the independent samples Student’s t-test.

Non-normally distributed or ordinal variables (e.g., OAA/S score, intubation score, infusion time, lignocaine dose, time to achieve BIS < 70, intubation time, number of intubation attempts) were compared using the Mann-Whitney U test.

Categorical variables (sex, ASA grade, airway characteristics, complications, and other clinical outcomes) were analyzed using the chi-square test or Fisher’s exact test when expected cell counts were <5.

Correlation between sedation indices (BIS and OAA/S scores) was assessed using Spearman’s rank-order correlation coefficient (ρ), with coefficients interpreted as moderate (0.5-0.8) or strong (>0.8).

A two-tailed p-value < 0.05 was considered statistically significant, and a p-value < 0.01 was considered highly significant.

## Results

A total of 40 consecutive patients who met the inclusion criteria were included in the analysis. One patient in the dexmedetomidine group did not proceed with AFOI due to procedural apprehension and was excluded from further analysis. The final study cohort comprised 19 patients in Group D and 20 patients in Group PF.

Baseline demographic characteristics and difficult airway parameters were comparable between the two groups (Table 1-2).

**Table 1:** Comparison of Patients’ Demographic Data Between the Two Groups. Overall Fisher’s exact test was used to calculate p-values. Data are presented as mean ± standard deviation or number of patients, as appropriate. t = independent t-test; χ² = chi-square test; ^$^Yates’ continuity correction and Fisher’s exact test. A two-tailed p < 0.05 was considered statistically significant. ASA, American Society of Anesthesiologists; BMI, body mass index; Group D, group dexmedetomidine; Group PF, group propofol-fentanyl; Stat. test, statistical test

Characteristics	Group D (n = 19)	Group PF (n = 20)	Stat. test	p-value
Age (years)	41.89 ± 11.04	39.15 ± 7.62	t = 0.90	0.375^$^
BMI (kg/m^2^)	25.65 ± 3.12	25.35 ± 3.12	t = 0.30	0.769^$^
Sex (males/females)	14/5	12/8	χ² = 0.45	0.501^$^
ASA grade (I/II)	15/ 4	15/5	χ² = 0.00	1.000^$^

**Table 2:** Comparison of Patients’ Difficult Airway Parameters Between the Two Groups. Overall Fisher’s exact test was used to calculate p-values. ^†^Fisher’s exact test; ^‡^Fisher-Freeman-Halton exact test. A two-tailed p < 0.05 was considered statistically significant. MMP, modified Mallampati score; TMD, thyromental distance; Group D, group dexmedetomidine; Group PF, group propofol-fentanyl

Characteristics	Group D (n = 19)	Group PF (n = 20)	p-value
Mouth opening (%) (<2.5/≤3) cm	21.2/78.8	20/80	>0.50^†^
MMP (3/4) (%)	84.2/15.8	80/20	1.000^†^
Neck extension <80° (yes/no)	84.2/15.8	80/20	1.000^†^
Cervical spine disease (%) (present/absent)	31.6/68.4	35/65.0	1.000^†^
TMD (%) (6.5/>6.5) cm	100/0	90/10	0.233^‡^

**Figure 1:**
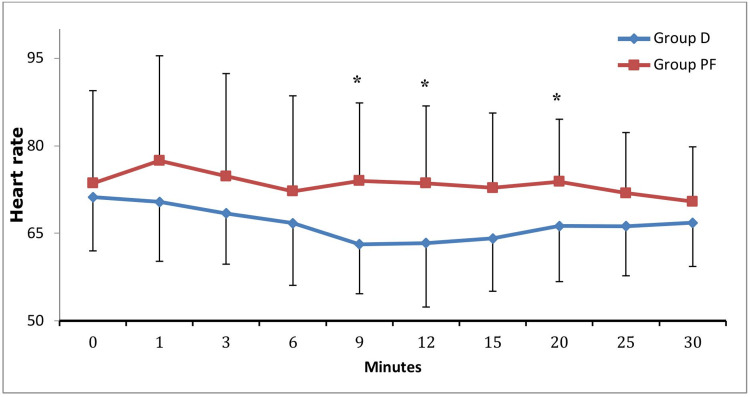
Heart Rate Comparing the Two Groups. Group D, group dexmedetomidine; Group PF, group propofol-fentanyl

**Figure 2:**
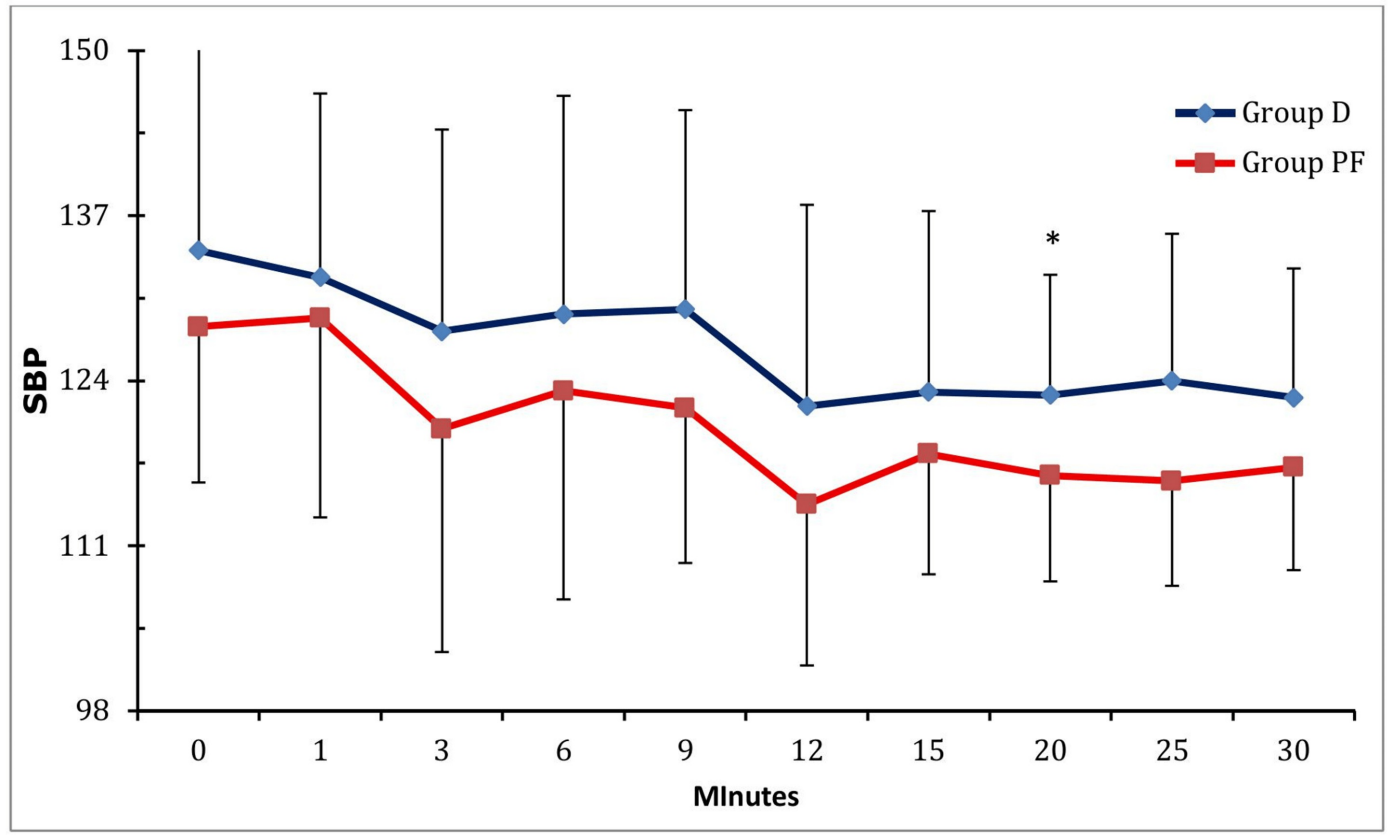
Comparison of SBP Between the Two Groups. Group D, group dexmedetomidine; Group PF, group propofol-fentanyl; SBP, systolic blood pressure (mmHg)

Group D demonstrated significantly lower heart rates compared to Group PF at nine, 12, and 20 minutes (Mann-Whitney U test; p < 0.05) (Table 3; Figure 1). SBP was lower in Group PF, with statistical significance achieved only at 20 minutes (p = 0.031) (Table 4; Figure 2). DBP, MAP, and SpO2 values demonstrated no significant differences between groups (p > 0.05).

**Table 3:** Comparison of Heart Rate (HR) Between the Two Groups. The Mann-Whitney U test was used to calculate p-values. *p < 0.05, statistically significant (Mann-Whitney U test); median (IQR (Q1-Q3)); Q1-Q3 = 25th and 75th percentile, respectively. Group D, group dexmedetomidine; Group PF, group propofol-fentanyl; IQR, interquartile range

HR (minute)	Group D			Group PF			Mann-Whitney U test	
	Median (/minute)	IQR (/minute)		Median (mmHg)	IQR (/minute)			
		Q1	Q3		Q1	Q3	Z	p
0	75.0	64.0	78.0	68.0	61.5	89.0	0.098	0.922
1	68.0	62.0	78.0	68.5	65.3	97.0	1.042	0.298
3	66.0	62.0	74.0	67.0	62.0	86.0	0.875	0.382
6	68.0	60.0	72.0	65.0	60.5	77.0	0.619	0.536
9	60.0	58.0	70.0	70.5	64.0	81.3	2.650	0.008*
12	65.0	52.0	72.0	72.0	65.3	79.0	2.266	0.023*
15	65.0	55.0	70.0	69.5	61.8	86.8	1.929	0.054
20	65.0	60.0	70.0	69.0	66.0	84.0	2.002	0.045*
25	65.0	60.0	72.0	69.0	64.0	80.0	1.592	0.111
30	65.0	60.0	72.0	69.0	62.5	80.0	1.114	0.265

**Table 4:** Comparison of Systolic Blood Pressure (SBP) Between the Two Groups. The Mann-Whitney U test was used to calculate p-values. *p < 0.05, statistically significant (Mann-Whitney U test); median (IQR (Q1-Q3)); Q1-Q3 - 25th and 75th percentile, respectively. Group D, group dexmedetomidine; Group PF, group propofol-fentanyl; IQR, interquartile range; SBP, systolic blood pressure (mmHg)

SBP (minute)	Group D			Group PF			Mann-Whitney U test	
	Median (mmHg)	IQR (mmHg)		Median (mmHg)	IQR (mmHg)			
		Q1	Q3		Q1	Q3	Z	p
0	133.0	127.0	145.0	130.0	118.5	134.5	1.411	0.158
1	130.0	122.0	148.0	127.5	122.0	138.5	0.690	0.490
3	124.0	118.0	135.0	119.0	107.8	132.0	1.591	0.112
6	136.0	116.0	143.0	122.0	117.8	134.0	1.196	0.232
9	130.0	115.0	141.0	123.5	115.0	129.8	1.549	0.121
12	122.0	110.0	136.0	114.0	102.0	123.3	1.649	0.099
15	124.0	112.0	134.0	116.0	110.5	125.0	0.929	0.353
20	124.0	116.0	130.0	116.0	110.0	123.3	2.159	0.031*
25	122.0	116.0	135.0	116.0	110.5	122.0	1.949	0.051
30	122.0	116.0	130.0	120.0	110.5	122.0	1.625	0.104

**Figure 3:**
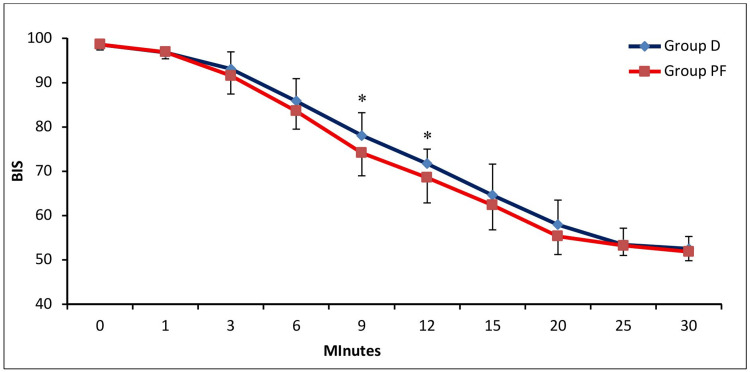
Comparison of BIS Between the Two Groups. BIS, Bispectral Index; Group D, group dexmedetomidine; Group PF, group propofol-fentanyl

Time to achieve BIS 70 was shorter in Group PF (10.00 ± 2.18 minutes) compared with Group D (11.89 ± 2.49 minutes) (Tables 5-6; Figure 3), with BIS values declining earlier in Group PF than Group D, showing statistically significant differences at nine and 12 minutes (Mann-Whitney U test; p = 0.018 and p = 0.049, respectively). Beyond the 15-minute time point, the rate of BIS decline was comparable between groups.

**Table 5:** Comparison of Bispectral Index (BIS) Between the Two Groups. The Mann-Whitney U test was used to calculate p-values. *p < 0.05, and is statistically significant (Mann-Whitney U test); median (IQR (Q1-Q3)), Q1-Q3 - 25th and 75th percentile, respectively. BIS, Bispectral Index; Group D, group dexmedetomidine; Group PF, group propofol-fentanyl; IQR, interquartile range

BIS (minute)	Group D			Group PF			Mann-Whitney U test	
	Median	IQR		Median	IQR			
		Q1	Q3		Q1	Q3	Z	p
0	98.0	98.0	100.0	98.0	98.0	100.0	0.166	0.868
1	97.0	96.0	98.0	97.0	96.0	98.0	0.235	0.814
3	93.0	90.0	97.0	92.0	90.0	94.0	1.278	0.201
6	84.0	84.0	89.0	85.0	80.0	86.0	0.899	0.369
9	78.0	74.0	82.0	73.0	70.0	77.5	2.363	0.018*
12	70.0	70.0	74.0	70.0	68.0	70.0	1.971	0.049*
15	70.0	60.0	70.0	60.0	58.0	70.0	1.108	0.268
20	58.0	55.0	60.0	55.0	52.0	58.0	1.686	0.092
25	54.0	51.0	55.0	54.0	51.0	55.0	0.470	0.638
30	52.0	50.0	55.0	52.0	50.0	53.0	0.727	0.467

**Table 6:** Comparison of Time Required to Reach BIS < 70 Between the Two Groups. A two-tailed test is used to calculate the p-value. *A two-tailed p < 0.05 was considered statistically significant. t = independent samples t-test. BIS, Bispectral Index; Group D, group dexmedetomidine; Group PF, group propofol-fentanyl; N, numbers; SD, standard deviation

Groups	N	Time to achieve BIS <70 (mean ± SD) (minutes)	t	p-value
Group D	19	11.89 ± 2.49	2.533	0.016*
Group PF	20	10.00 ± 2.18		

**Figure 4:**
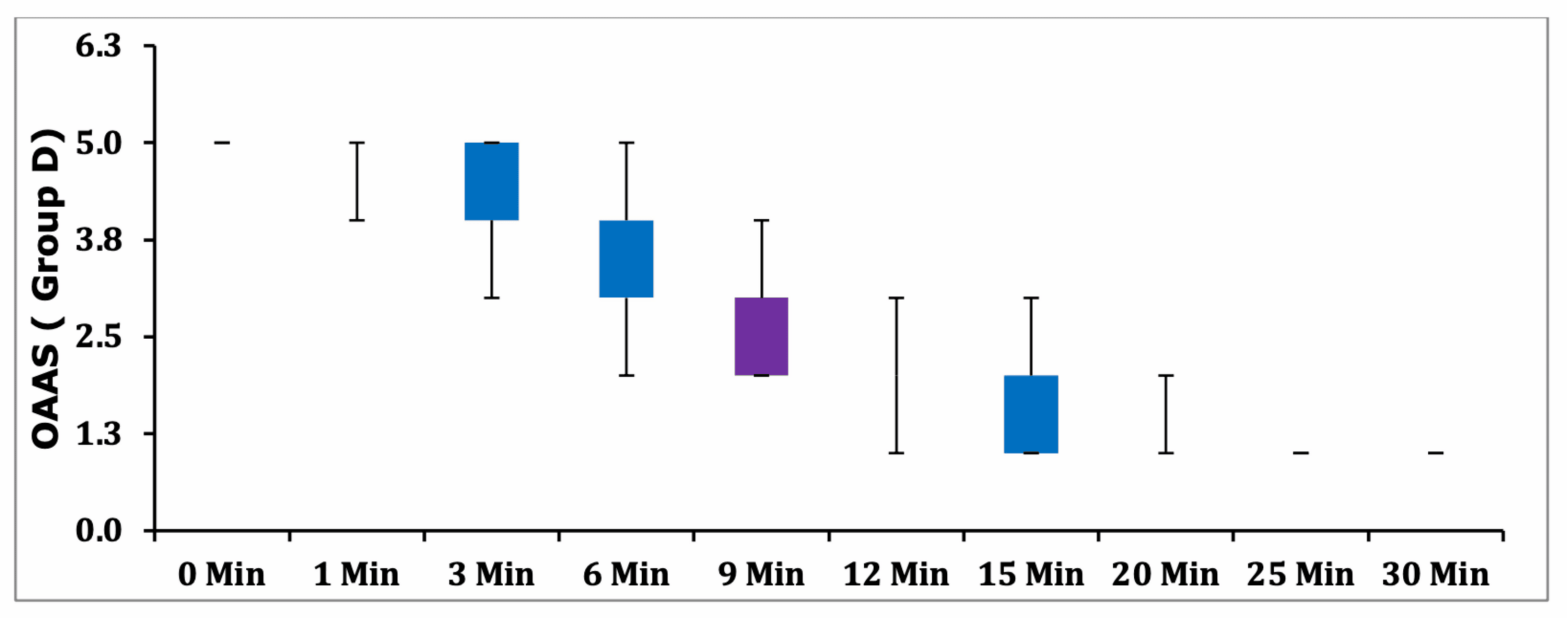
Box Plot Graph - OAA/S in Group D. Group D, group dexmedetomidine; OAA/S, Observer’s Assessment of Alertness/Sedation Scale

**Figure 5:**
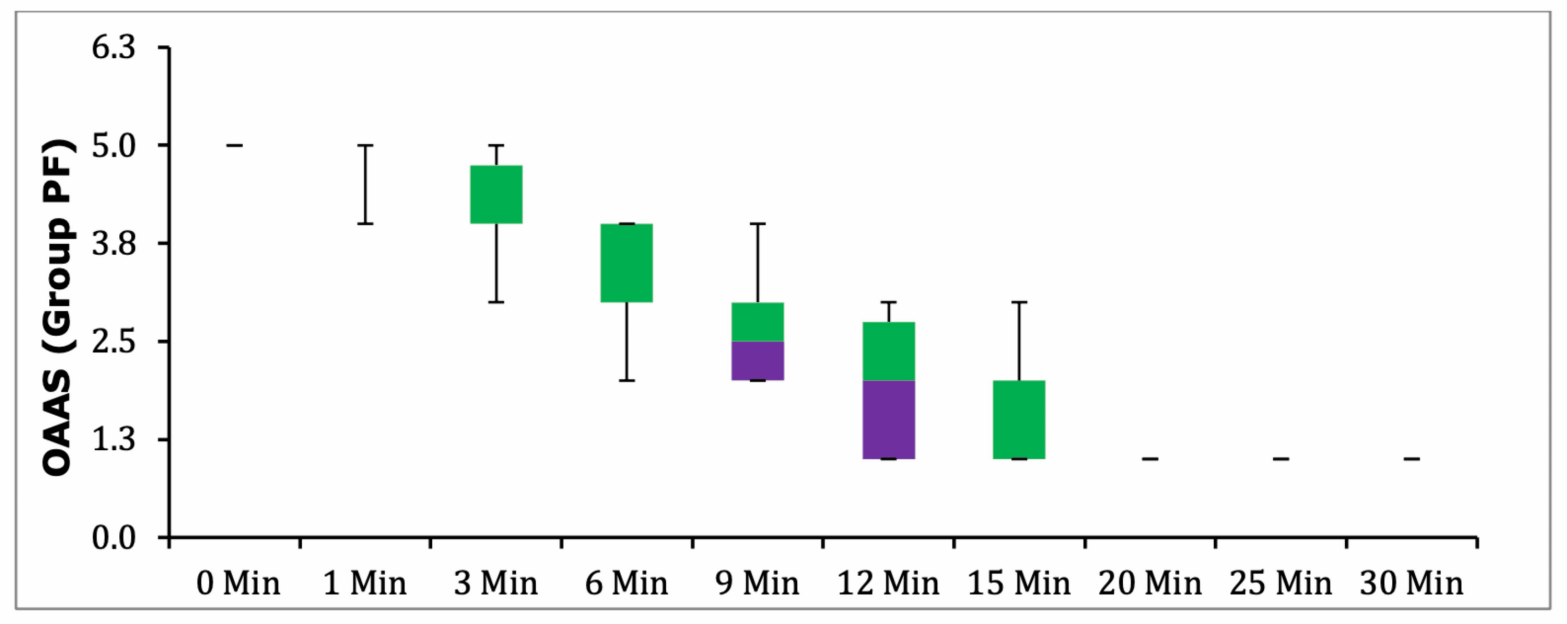
Box Plot Graph - OAA/S in Group PF. BIS, Bispectral Index; Group D, group dexmedetomidine; Group PF, group propofol-fentanyl; OAA/S, Observer’s Assessment of Alertness/Sedation Scale

Box-plot analysis of OAA/S scores demonstrated a steeper decline in sedation levels in Group PF relative to Group D; however, these differences did not reach statistical significance at any measured time point (p > 0.05) (Figures 4-5).

Spearman's rank correlation analysis evaluated the relationship between BIS monitoring and OAA/S assessment. Group D demonstrated significant correlations at three, six, nine, 12, 15, and 20 minutes; Group PF showed significant correlations at three, nine, 12, and 15 minutes (Table 7).

**Table 7:** Spearman Rank Correlation Between the BIS and OAA/S Between the Two Groups. *p < 0.05 = considered statistically significant. BIS, Bispectral Index; Group D, group dexmedetomidine; Group PF, group propofol-fentanyl; OAA/S, Observer’s Assessment of Alertness/Sedation Scale

Time (minute)	Group D		Group PF	
	Spearman's rho - ρ	p-value	Spearman's rho - ρ	p-value
0	-	-	-	-
1	0.226	0.351	0.120	0.614
3	0.881	<0.001*	0.749	<0.001*
6	0.557	0.013*	0.437	0.054
9	0.667	0.002*	0.900	<0.001*
12	0.610	0.006*	0.777	<0.001*
15	0.846	<0.001*	0.815	<0.001*
20	0.570	0.011*	-	-

**Figure 6:**
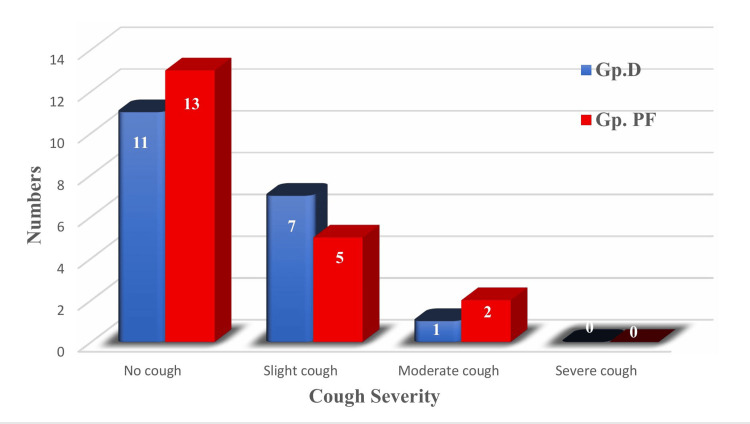
Comparison of Cough Severity Between the Two Groups. Group D, group dexmedetomidine; Group PF, group propofol-fentanyl

Cough severity was similar between the groups (p = 0.668, i.e., p > 0.05; Pearson chi-square test) and was considered non-significant. No cough %(n) occurred in 57.9 (11) of Group D and 65.0 (13) of Group PF; slight cough in 36.8 (7) vs. 25.0 (5); moderate cough in 5.3 (1) vs. 10.0 (2). None of the patients in either group exhibited a severe cough (Figure 6).

Although the anesthesia quality and vocal cord relaxation demonstrated superior characteristics in Group PF, they did not achieve statistical significance. However, immediate ETT tolerance differed significantly between groups (p = 0.047), with superior scores in Group PF compared with Group D. Thereby, the composite intubation score was significantly higher in Group PF than Group D (7.11 vs. 7.8; p = 0.039), indicating superior intubating conditions (Tables 8-9), although both groups had an easy grade of AFOI [15].

**Table 8:** Comparison of Patient Intubation Scores Between the Two Groups. Overall Fisher’s exact test was used to calculate p-values. ^†^Yates’ continuity-corrected chi-square values. ^$^Fisher’s exact test was used to calculate p-values. *A two-tailed p < 0.05 was considered statistically significant. Group D, group dexmedetomidine; Group PF, group propofol-fentanyl; ETT, endotracheal tube; N/(%), number/percentage; Stat. test, statistical test

Criteria	Evaluation	Score	Group D N (%)	Group PF N (%)	Stat. test	p-value
Anesthesia quality	Asleep, deep sedation	2	19 (100)	20 (100)	χ² = 0.00^†^	1.000^$^
	Slight, resistance	0	0	0		
Vocal cords relaxation	No	0	0	0	χ² = 3.42^†^	0.064^$^
	In part, middle	1	3 (15.8)	0		
	Relaxed, open	2	16(84.2)	20 (100)		
ETT tolerance	Bad, disturbing - cough, swallowing	0	0	0	χ² = 6.12^†^	0.047^$^
	Middle - coughing or swallowing not disturbing the procedure	2	7 (36.8)	2 (10)		
	Good, no coughing or swallowing	4	12 (63.2)	18 (90)		

**Table 9:** Comparison of Total Intubation Score Between the Two Groups. The Mann-Whitney U test was used to calculate p-values. Values are expressed as mean ± standard deviation. t = independent samples t-test; *p < 0.05 and is considered statistically significant (Mann-Whitney U test/p-value test). Group D, group dexmedetomidine; Group PF, group propofol-fentanyl

Groups	N	Total intubation score mean ± SD	t	p-value
Group D	19	7.11 ± 1.24	2.230	0.032*
Group PF	20	7.80 ± 0.62		

Intubation time was shorter in Group PF (16.10 ± 10.95 seconds) than in Group D (27.79 ± 21.18 seconds; p = 0.036) (Table 10).

**Table 10:** Comparison of Total Intubation Time Between the Two Groups. Student’s independent t-test was used to calculate p-values. *p < 0.05: considered statistically significant (t - t-test). Values are expressed as mean ± standard deviation (SD); t = independent t-test. Group D, group dexmedetomidine; Group PF, group propofol-fentanyl

Groups	N	Intubation time (mean ± SD) (seconds)	t	p-value
Group D	19	27.79 ± 21.18	2.181	0.036*
Group PF	20	16.10 ±10.95		

First-attempt intubation success rates were comparable between groups, 94.7% vs. 95% in Group D and Group PF, respectively (p = 0.970). One patient in both groups was intubated on the second attempt.

Airway complications (SpO2 < 95%) occurred in 10.5% of Group D (n = 2) and 15.0% of Group PF (n = 3), all resolving with basic airway maneuvers; two Group PF patients required adjunctive bag-mask ventilation (p = 0.676). Fiberoptic bronchoscopy procedures were completed successfully in all patients without abandonment in either group.

Bradycardia (defined as heart rate < 50 bpm) occurred exclusively in Group D (n = 2/19; heart rates 45 and 43 bpm), one patient required intravenous glycopyrrolate 0.2 mg, and in none of the patients in Group PF (n = 0/20). Although the incidence was numerically higher in Group D than in Group PF but was not statistically significant, p = 0.23 (Fisher’s exact test). 

Postoperative sore throat occurred in two Group D patients and none in Group PF (p = 0.136, Fisher’s exact test) (Table 11). One case was associated with a second intubation attempt; the other followed a single uncomplicated intubation. Both patients received treatment with 3% hypertonic saline nebulization, with complete symptom resolution by postoperative day 2. No intra-procedural recall of the procedure was reported in either group.

**Table 11:** Comparison of the Postoperative Sore Throat Between the Two Groups. Fisher’s exact test was used to calculate p-values. ^#^p > 0.05%, statistically not significant (Fisher’s exact test). Group D, group dexmedetomidine; Group PF, group propofol-fentanyl; N/(%), number/percentage

Postoperative discomfort	Category		Total N/(%)	p-value^#^
	Group D N/(%)	Group PF N/(%)		
Yes	2/(10.5)	0/(0.0)	2/(5.1)	0.136
No	17/(89.5)	20/(100.0)	37/(94.9)	
Total	19/(100.0)	20/(100.0)	39/(100)	

Lidocaine requirements were similar between groups (Group D: 4.75 ± 1.40 mg/kg vs. Group PF: 4.42 ± 1.24 mg/kg; p = 0.442). Mean doses of propofol were 85.50 ± 21.64 mg, fentanyl - 76.50 ±13.29 µg, and dexmedetomidine - 83.68 ± 17.55 µg. None of the patients in either group required conversion of AFOI into intubation under GA.

## Discussion

In this prospective comparative observational study involving neurosurgical patients with anticipated difficult airways, the propofol-fentanyl combination provided superior immediate ETT tolerance, higher composite intubation scores, shorter intubation time, and fewer postoperative complications, such as sore throat, compared with dexmedetomidine, while both regimens achieved adequate conscious sedation for orotracheal AFOI using the SAGO technique.

AFOI requires balanced sedation, airway reflex suppression, patient cooperation, and preservation of spontaneous ventilation. Previous studies have demonstrated improved airway reflex attenuation and procedural acceptability with propofol-opioid combinations, whereas dexmedetomidine offers cooperative sedation with stable respiration but dose-dependent variable intubation conditions [23-28]. While fentanyl-midazolam and dexmedetomidine-based regimens have been associated with favorable airway preservation and hemodynamic profiles [23,24], the present study observed more favorable composite intubation scores with propofol-fentanyl combination, likely reflecting enhanced hypnosis and analgesia achieved through multimodal sedation.

Differences in reported intubation conditions across studies may reflect heterogeneity in airway routes, topicalization techniques, assessment methods, and drug dosing. In a systematic review and meta-analysis by El-Boghdadly et al. [8], they identified dexmedetomidine as a useful agent for awake tracheal intubation but noted the limitation of prolonged loading infusion. In the present study, both dexmedetomidine and propofol-fentanyl were administered using an identical standardized 10-minute loading period, allowing direct comparison while minimizing infusion-related variability. The present study used a composite intubation score (anesthesia quality, vocal cord relaxation, immediate ETT tolerance) and cough severity, enabling comprehensive assessment in a heterogeneous difficult airway population. Similar findings favoring fentanyl-based regimens have been reported with structured scoring systems [29,30], whereas studies supporting dexmedetomidine often used different outcome measures or monotherapy comparisons [21]. This suggests that observed differences in intubation conditions were more likely related to the sedative regimens rather than differences in drug administration protocols.

Comparable airway tolerance across sedative techniques has been reported previously. Lallo et al. [19] found no significant difference in cough severity between propofol and remifentanil, while Bonnin et al. [20] reported similar intubation conditions with propofol TCI and inhaled sevoflurane, emphasizing the importance of protocolized airway management rather than reliance on a single agent.

Intubation times in the present study were shorter than in some earlier reports [22], likely reflecting the use of a standardized airway management protocol incorporating structured sedation delivery, the SAGO technique, and uniform assessment of intubation conditions. Although some studies have reported improved intubation efficiency with dexmedetomidine under specific conditions [31], heterogeneity in airway techniques, sedation targets, and operator experience limits direct comparison across studies. Ma et al. [32] reported shorter intubation times using a structured protocol with regional airway blocks in simulated difficult airways, further supporting the concept that protocol-driven airway preparation and sedation strategies, rather than the sedative agent alone, are key determinants of intubation efficiency during AFOI.

High first-attempt success rates were achieved in both groups, indicating that both sedative strategies were effective for AFOI when applied within a structured protocol. Variability in intubation efficiency reported across studies has been attributed to differences in sedation regimens and airway techniques; for example, Cattano et al. [9] reported more frequent repeat intubation attempts with dexmedetomidine compared with remifentanil, whereas Ma et al. [32] reported a first-attempt success rate comparable to that observed in the present study. These findings suggest that high first-attempt success is more closely related to standardized airway topicalization, structured sedation monitoring, and operator experience than to the sedative agent alone.

The time to achieve adequate sedation (BIS < 70) was shorter in the propofol-fentanyl group, indicating a faster onset of effective sedation. BIS identified acceptable sedation earlier than the corresponding OAA/S score, consistent with previous observations that electrophysiological monitoring may precede clinical sedation endpoints depending on the agent used [13]. Slower attainment of equivalent clinical sedation with dexmedetomidine has also been reported by Cattano et al. [9], where remifentanil achieved target sedation more rapidly. Although remifentanil and fentanyl differ in pharmacokinetic and pharmacodynamic profiles, these findings support the broader concept that opioid-based sedation may facilitate favorable intubation conditions and highlight the relatively slower onset associated with dexmedetomidine.

Despite reported variability in the correlation between BIS and clinical sedation scales across different sedatives [13], a strong correlation between BIS and OAA/S scores was observed in both groups. The combined use of EEG-based monitoring and clinical sedation scales provided complementary assessment of sedation depth, facilitated consistent attainment of target conscious sedation, and may have contributed to improved procedural conditions and patient tolerance during AFOI.

Hemodynamic trends were consistent with the known pharmacological profiles of the agents. Dexmedetomidine was associated with relatively stable blood pressure, whereas the Group PF showed lower values, with a significant difference observed only in SBP at the twentieth minute. These findings align with previous reports describing the sympatholytic effects of dexmedetomidine and the mild hypotensive effects of propofol and opioids [8,25,26], possibly reflecting reduced sympathetic response following airway instrumentation and transition toward general anesthesia.

Respiratory events were infrequent in both groups, although brief episodes of apnea or oxygen desaturation occurred slightly more often with propofol-fentanyl, consistent with the known respiratory depressant effects of these agents [8]. In contrast, dexmedetomidine better preserved spontaneous ventilation, as previously reported [7,23]. All events were promptly recognized and managed. Dexmedetomidine-related adverse effects such as bradycardia have been described, particularly at higher doses [10]. In this study, routine premedication with intramuscular glycopyrrolate may have reduced clinically significant bradycardia; however, two patients in the Group D developed bradycardia, one requiring additional intravenous glycopyrrolate. These findings highlight the importance of continuous monitoring during AFOI irrespective of the sedative regimen used.

Post-intubation airway-related morbidity was minimal in both groups. Two patients in the dexmedetomidine group reported sore throat, which resolved with conservative management, while none in the propofol-fentanyl group reported similar symptoms. These findings are comparable to previous studies reporting low rates of airway discomfort with different sedative techniques during AFOI [19,20,22,32]. Improved ETT tolerance and reduced coughing in the PF group may have contributed to the lower incidence of sore throat observed.

Procedural recall, which has been variably reported with propofol, opioid, and dexmedetomidine-based sedation [19,22,26], was not observed in the present study. This absence of recall may be attributable to careful sedation titration guided by both BIS and OAA/S monitoring, standardized airway topicalization, and the exclusive use of the orotracheal route. Although dexmedetomidine has been associated with lower recall in some studies [26], patient tolerance and procedural acceptability in the present cohort were more favorable with the propofol-fentanyl combination, suggesting that sedation strategy and monitoring may be as influential as the choice of sedative agent.

The standardized use of the SAGO technique avoided invasive airway blocks and likely contributed to improved patient tolerance and smoother intubating conditions, as supported by other authors [19,20].

In this study, propofol-fentanyl combination was found to be an effective and safe sedative regimen for AFOI, providing satisfactory sedation, analgesia, patient comfort, acceptable intubating conditions, and hemodynamic stability. Although dexmedetomidine offers certain clinical advantages, propofol with fentanyl remains a dependable and widely accessible option, particularly where rapid titration and clinician familiarity are essential. The present study is strengthened by a protocolized design incorporating BIS-guided sedation, standardized intubation and tolerance scoring, and the use of the SAGO technique for airway topicalization, which avoided the discomfort of invasive airway blocks and likely contributed to improved patient tolerance and smoother intubating conditions. These methodological features enhance the clinical relevance and applicability of the findings compared with earlier studies relying on heterogeneous populations and predominantly subjective assessments.

Limitations and future directions

This study has a few limitations. Its single-center observational design and small sample size limit the precision and generalizability of the findings. The open-label design and anesthesiologist-directed selection of sedation regimen may have introduced observer bias, particularly in subjective assessments. Inclusion of only elective neurosurgical patients with anticipated difficult airways limits applicability to emergency settings. Although all intubations were performed by a single experienced anesthesiologist to maintain procedural consistency, this may reduce external validity. Larger multicenter randomized controlled trials involving multiple operators are needed to validate these findings and improve generalizability.

## Conclusions

In conclusion, the propofol-fentanyl combination provided safe and effective sedation for awake fiberoptic orotracheal intubation, with better ETT tolerance, higher composite intubation scores, shorter intubation times, and fewer postoperative complications compared with dexmedetomidine. While dexmedetomidine offers cooperative sedation with respiratory stability, propofol-fentanyl remains a reliable and widely available alternative, particularly when rapid titration and clinician familiarity are important. BIS-guided sedation and standardized airway topicalization enhance the clinical applicability of these findings; however, larger randomized controlled trials are needed to confirm these results and support individualized sedative selection in anticipated difficult airways.
